# Gene signature of m^6^A RNA regulators in diagnosis, prognosis, treatment, and immune microenvironment for cervical cancer

**DOI:** 10.1038/s41598-022-22211-2

**Published:** 2022-10-21

**Authors:** Shizhi Wang, Bo Ding, Shiyuan Wang, Wenjing Yan, Qianqian Xia, Dan Meng, Shuqian Xie, Siyuan Shen, Bingjia Yu, Haohan Liu, Jing Hu, Xing Zhang

**Affiliations:** 1 Key Laboratory of Environmental Medicine Engineering, Ministry of Education, School of Public Health, Southeast University, 87 Dingjiaqiao, Gulou District, Nanjing, 210009 China; 2 Department of Gynecology and Obstetrics, School of Medicine, Zhongda Hospital, Southeast University, Nanjing, China

**Keywords:** Cancer, Diseases, Medical research

## Abstract

Continuing studies imply that m^6^A RNA modification is involved in the development of cervical cancer (CC), but lack strong support on recurrence and diagnosis prediction. In this research, a comprehensive analysis of 33 m^6^A regulators was performed to fulfill them. Here, we performed diagnostic and prognosis models and identified key regulators, respectively. Then the CC patients were separated into two clusters in accordance with 33 regulators, and participants in the cluster 1 had a worse prognosis. Subsequently, the m^6^AScore was calculated to quantify the m^6^A modification pattern based on regulators and we found that patients in cluster 1 had higher m^6^AScore. Afterwards, immune microenvironment, cell infiltration, escape analyses and tumor burden mutation analyses were executed, and results showed that m^6^AScore was correlated with them, but to a limited extent. Interestingly, HLAs and immune checkpoint expression, and immunophenoscore in patients with high-m^6^AScores were significantly lower than those in the low-m^6^AScore group. These suggested the m^6^AScores might be used to predict the feasibility of immunotherapy in patients. Results provided a distinctive perspective on m^6^A modification and theoretical basis for CC diagnosis, prognosis, clinical treatment strategies, and potential mechanism exploration.

## Introduction

Cervical cancer (CC), which is currently the fourth most common malignancy in women worldwide, led the cause of malignant tumor deaths and a heavy social burden in developing countries^1^. Although early screening and effective interventions can prevent the occurrence of CC and improve the prognosis of CC, the situation is still severe, such as the high recurrence rate of CC. Apart from the recognized factor of HPV infection, its pathogenesis is not fully understood^2,3^. Given the deleterious influence of CC, efforts are needed to explore the potential biomarkers for the diagnosis and prognosis, as well as feasible treatment strategies.

N6-methyladenosine (m^6^A) RNA methylation is the most common conserved internal transcriptional and modification epigenetic modification. m^6^A is a dynamic process, and three kinds of essential regulators (known as “writer”, “eraser”, and “reader”) are involved in the regulation of this modification process in the human body, leading to several facets changes in RNA processing, including RNA stability, alternative splicing and translation^4–11^. As a hot spot in epigenetic research in recent years, the fundamental role of m^6^A in cancer development and prognosis may help us clarify the mechanism of CC with a novel perspective.

A growing body of literature studies has shown that the imbalance of m^6^A modification regulators affects a series of biomolecular events by influencing target RNAs, and ultimately affecting the occurrence, development, and prognosis of many diseases including cancers. Evidence indicates that upregulation induced by m^6^A methylation could contribute to the increased cancer stemness cell in colon cancer^12^. In addition, the abnormal expression of methyltransferase complex components in m^6^A modification affects both gastric cancer and liver cancer, including adjusted by FTO and WTAP instead of METTL3 and METTL14, which were considered as principal roles in m^6^A modification^13,14^. Collectively, although the mechanism of m^6^A modification in cancer including CC has been well studied in recent years^15,16^, the evidence for the importance of m^6^A modification importance in CC recurrence and diagnostic analysis is still lacking.

In this study, we used CC patient data from GEO and TCGA to comprehensively analyze 33 m^6^A regulators and their indicative roles for both CC diagnosis and prognosis. Essential targets were identified by constructing diagnostic and prognostic models of CC, and drug sensitivity analysis was carried out based on these factors. We also subsequently assessed the potential functions of m^6^A RNA regulators through immune prediction and enrichment analyses, and explored the clinical treatment strategies of CC.

## Materials and methods

### Ethical conduct of research

The authors pointed out that the Ethics Committee of Southeast University approved this study, and the informed consent was acquired from each participant recruited and all samples were used in compliance with the institution’s ethical regulations. The research design was in accordance with the Declaration of Helsinki.

### Data resource

The transcriptome sequencing data (read counts and FPKM normalized) of 306 CC and three normal patients were downloaded from The Cancer Genome Atlas (TCGA) portal (https://tcga-data.nci.nih.gov/tcga/) and transformed obtained gene expression values into per kilobase million (TPM) values. The expressions of m^6^A modification regulators in patients from the data matrix were extracted for the subsequent analysis. The CC clinical information was downloaded from the TCGA portal. The GSE63514 and GSE6791 data were downloaded from the Gene Expression Omnibus (GEO) database (https://www.ncbi.nlm.nih.gov/gds).

### Selection of m^6^A RNA methylation regulators

We first aimed to m^6^A modification regulators from published literature^17–19^. Subsequently, we extracted the expression levels of regulators from the TCGA and cervical tissue transcription data, deleting the regulators in which expression levels were unavailable. Finally, 33 m^6^A modification regulators were selected as candidate molecules for this study^16,20^ (Table S1).

### Significant differential expression gene (DEGs) analysis

To screen the regulators which play an essential role in the development of CC, differential expression analysis was performed using the R program with the “DESeq2” package. Among all the gene analyzed, FDR (false discovery rate) < 0.05 were considered as a criterion and the DEGs were obtained for subsequent analysis. Gene copy number variation (CNV) analysis was shown by cBioportal tools (http://www.cbioportal.org/). Venn plot was displayed by the venny 2.1 tool (https://bioinfogp.cnb.csic.es/tools/venny/).

### Consensus clustering for subgroups identification

To investigate the function of regulators in CC, we clustered cancer tissues into two subgroups by R program with the “ConsensusClusterPlus” package. Then the principal component analysis (PCA) analysis and t-distributed stochastic neighbor embedding (t-SNE) were utilized to study the gene expression patterns in different CC subgroups. Furthermore, Kaplan–Meier analysis was drawn to assess prognosis between subgroups and compared using the log-rank test.

### Pathway analysis and acquisition of gene sets

Gene Set Cancer Analysis (GSCA, bioinfo.life.hust.edu.cn/web/GSCALite/) database was used to find clues about m^6^A regulators in biological processes. GSEA analysis of patients in two clusters was performed using c2.cp.kegg.v7.4.symbols.gmt and c5.go.v7.4.symbols.gmt downloaded from the Gene Set Enrichment Analysis (GSEA, https://www.gsea-msigdb.org/gsea/index.jsp) database. Then, the gene sets of interest were downloaded from the GSEA database, including BIOCARTA_CELLCYCLE_PATHWAY (M17770), BIOCARTA_CASPASE_PATHWAY (M17902), REACTOME_PYROPTOSIS (M41805) and WP_FERROPTOSIS (M39768). Moreover, the ferroptosis-related gene list was also obtained from the FerrDb database (http://www.zhounan.org/ferrdb/). The genes related to programmed necroptosis^21^ and cuproptosis^22^ were identified from literature reports.

### Identify important molecular markers through machine learning

To further accurately identified the critical m^6^A regulators affecting the diagnosis of CC, we used machine learning methods to construct a diagnostic model of CC, ranked the variables according to their importance, and visualized results by the R program.

The random forest (RF) model is a typical classifier that containing many decision trees. Patients with replacements were randomly selected from the initial dataset to assemble a sub-dataset. In this study, most of the 54 patients (28 cases and 24 normal control) from GSE63514 were used as a training set, while left participants were analyzed as a validation set. Based on the seed number 51, ROC was used to fit the optimal model using the most considerable value, and the final value selected for the RF model was mtry = 2. The fivefold cross-validation method was also applied during the analysis. RF was executed by the “randomForest” package.

The Support Vector Machines (SVM) model is an algorithm that is widely used in binary and multiple classifications. SVM core function can convert samples that were inseparable in low-dimensional into high-dimensional separable space to achieve better grouping. In this study, the tuning parameter ‘sigma’ was held constant at a value of 0.01744768, and the SVM model was performed by the “e1071” package of R.

Artificial Neural Network (ANN) is a complex network structure formed by interconnecting a series of treating units, which has been proved to be scientific and accurate in disease prediction in recent years^23–25^. In this study, we utilized the common regulators selected by RF and SVM as the input layer to construct an ANN model. Feedforward neurons generated a backpropagation during the training process, and the error rate of this backpropagation reflected the discrepancy between the model judgment and the actual patient status. During data transfer and processing, one output was produced, the result of the classification. After adjusting the weight of the input data, with the appropriate back-propagation error range as evidence, the best classification method was finally achieved. A total of 28 patients and 24 normal control from GSE63514 were used as a training set, while 19 cases and nine normal participants from GSE6791were analyzed as a validation set. The R packages “neuralnet” and “NeuralNetTools” were applied in this process.

### Construction and validation of the LASSO Cox regression algorithm

To study the prognostic value of m^6^A RNA methylation regulators, a univariate Cox regression analysis was implemented on the regulators for subsequent model construction. Firstly, Two-thirds of the samples were randomly selected as a training set for the establishment of the model, and the remaining samples were used for model reliability verification. Secondly, the LASSO Cox regression algorithm was implemented to develop a potential risk signature. Finally, we calculated the LASSO-risk score for a signature using the following formula:

$${\text{LASSO-risk score}}={\sum_{{\text{i}}=1}^{\text{n}}}{{\text{Coef}}_{\text{i}}}\times {\text{x}}_{\text{i}}.$$

Among them, coef represents the coefficients and x_i_ represents the relative expression value after the z-score transformation of the original expression value of each gene. In this study, this formula was used to calculate the risk score for each CC patient concerning OS and RFS, respectively. Furthermore, receiver operator characteristics (ROC) curves and area under the curve (AUC) were generated for signature validation using the R program with the “survivalROC” package, and the AUC was calculated for prediction evaluation.

The nomogram was constructed to evaluate the prediction probability of 2-, 3- and 5-year OS or RFS. The calibration curves show the 2-, 3- and 5-year OS or RFS were drawn to visualize the observed probabilities against the nomogram prediction. The R package “RMS” presented the nomogram and calibration curves. Decision curve analysis (DCA) with 2-, 3- and 5-year was performed to evaluate the suitability of the constructed model for clinical application by the “ggDCA” package.

### RNA expression detection and quantitative polymerase chain reaction (qPCR)

To determine the key m^6^A regulators (RBM15, HNRNPA2B1, NSUN2, RBMX, CBLL1, METTL3, YTHDF3, and ZC3H13) expression pattern in CC, a total of 20 fresh CC tissue and 20 paired adjacent non-tumor tissues were acquired from patients between August 2020 and September 2021 at Zhongda Hosptial and Nanjing Maternity (Table S2). All the samples were stored well at − 80 °C with treatment by RNAlaterTM Stabilization Solution (AM7021, Thermo Fisher, US). RNA extraction protocol was described in the previous literature published^26^, and the primer sequences involved were listed in Table S3.

### Generation of geneset scores based on PCA analysis

PCA was performed using the expression values of the 17 m^6^A regulators in all CC patients. Among them, both the first and second principal components were selected to participate in the calculation of m^6^AScore. In this study, in addition to m^6^AScore, a similar method was used to construct scores based on different genesets in the subsequent exploration process for comprehensive correlation analysis.

### Immune correlation analysis among groups

Estimation of STromal and Immune cells in MAlignant Tumor tissues using Expression data (ESTIMATE) algorithm was executed to calculate the immune score, stromal score, estimated score, and tumor purity of each CC patient based on m^6^A regulators. Cell-type Identification By Estimating Relative Subsets of RNA Transcripts (CIBERSORT, http://cibersort.stanford.edu/) was utilized to calculate the abundance of immune cells. The Tumor Immune Dysfunction and Exclusion (TIDE) algorithm (http://tide.dfci.harvard.edu/) was used to infer clinical response to immunotherapy in CC with m^6^A regulators expression profiles. The Cancer Immunome Atlas (TCIA, https://www.tcia.at/) database was utilized for downloading the immune checkpoint inhibitor (ICI) information and immunophenoscore (IPS), an index widely used to represent the immunogenicity.

### Drug sensitivity analysis

The Connectivity Map (CMap) database (https://portals.broadinstitute.org/cmap/) was utilized to list potential chemotherapeutic drugs and the half-maximal inhibitory concentration (IC50) was assessed to estimate the drug sensitivity using the “pRRophetic” package.

### Bioinformatic analysis

The following PPI (Protein–protein interaction) network was analyzed using the STRING tool (http://www.string-db.org/). Correlation analysis was performed to explore the association between m^6^A regulators and other interested genes obtained from the TCGA-CESC database. |Cor|> 0.3 was defined as a significant criterion. PPI network and correlation network were visualized by Cytoscape v3.9.1.

### Statistical analysis

All the statistical analyses were developed by R software (v4.0.5) and GraphPad Prism (v8.0.2). Spearman correlation analysis was calculated between m^6^A regulators and target elements. Kaplan–Meier survival analysis with log-rank test was performed to compare patients in different subgroups. The visualization of results was accomplished by R software and GraphPad Prism. *P* < 0.05 was considered statistically significant unless otherwise marked.

## Result

### A machine-learning diagnostic model derived m^6^A regulators in CC

**Figure 1:**
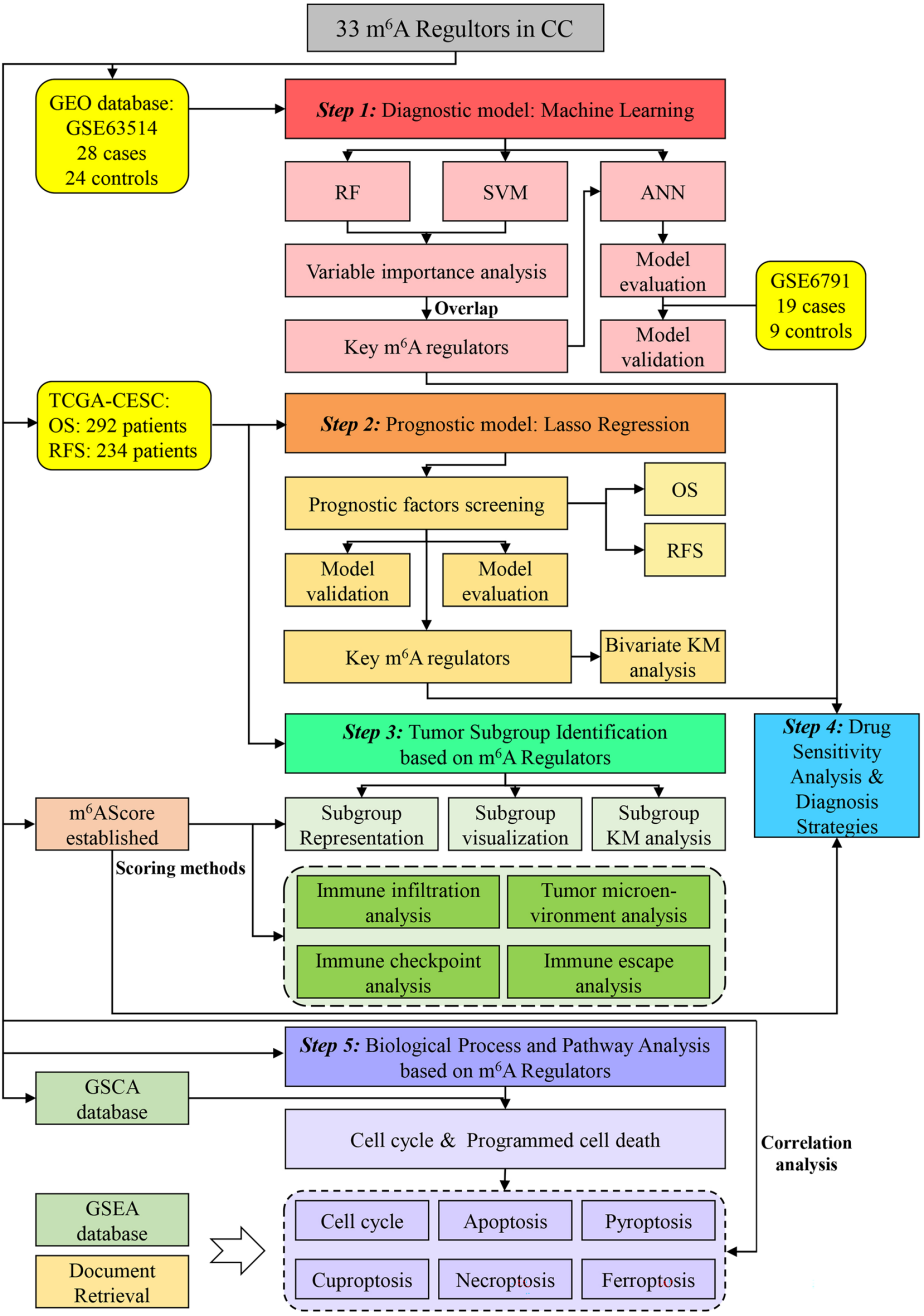
The flowchart of this study. *CC* cervical cancer, *RF* Random Forest, *SVM* support vector machines, *ANN* artificial neural networks, *OS* overall survival, *RFS* recurrence-free survival, *KM* Kaplan–Meier; *TCGA* The Cancer Genome Atlas, *GEO* Gene Expression Omnibus, *GSCA* Gene Set Cancer Analysis, *GSEA* Gene Set Enrichment Analysis.

All the bioinformatics analyses utilized in this study were executed as a flowchart in Fig. 1. The 33 selected m^6^A regulators were shown in Table S1. Before commencing the analyses, we constructed protein–protein Interaction (PPI) network and correlation network to investigate the associations between 33 m^6^A regulators, and the results showed that there were high functional interactions (minimum required interaction score > 0.4; Fig. S1a) among them. The CNVs analysis showed that IGF2BP2, FXR1, and NSUN2 had higher amplification frequencies, while ZC3H13 had a higher CNV deletions probability (Fig. S1b). Therefore, the expression correlations among 33 regulators demonstrated their close relationship (|R|> 0.2, *P* < 0.05; Fig. S1c). In addition, to explore the aberrant expression of m^6^A regulators in CC, we compared cancer patients and normal controls in TCGA, GSE63514 and GSE6791 datasets. As Fig. S1d showed, HNRNPA2B1, YTHDF2, RBM15, and NSUN2 were consistently up-regulated in tumor tissues in TCGA-CESC, GSE63514, and GSE6791 datasets.

**Figure 2:**
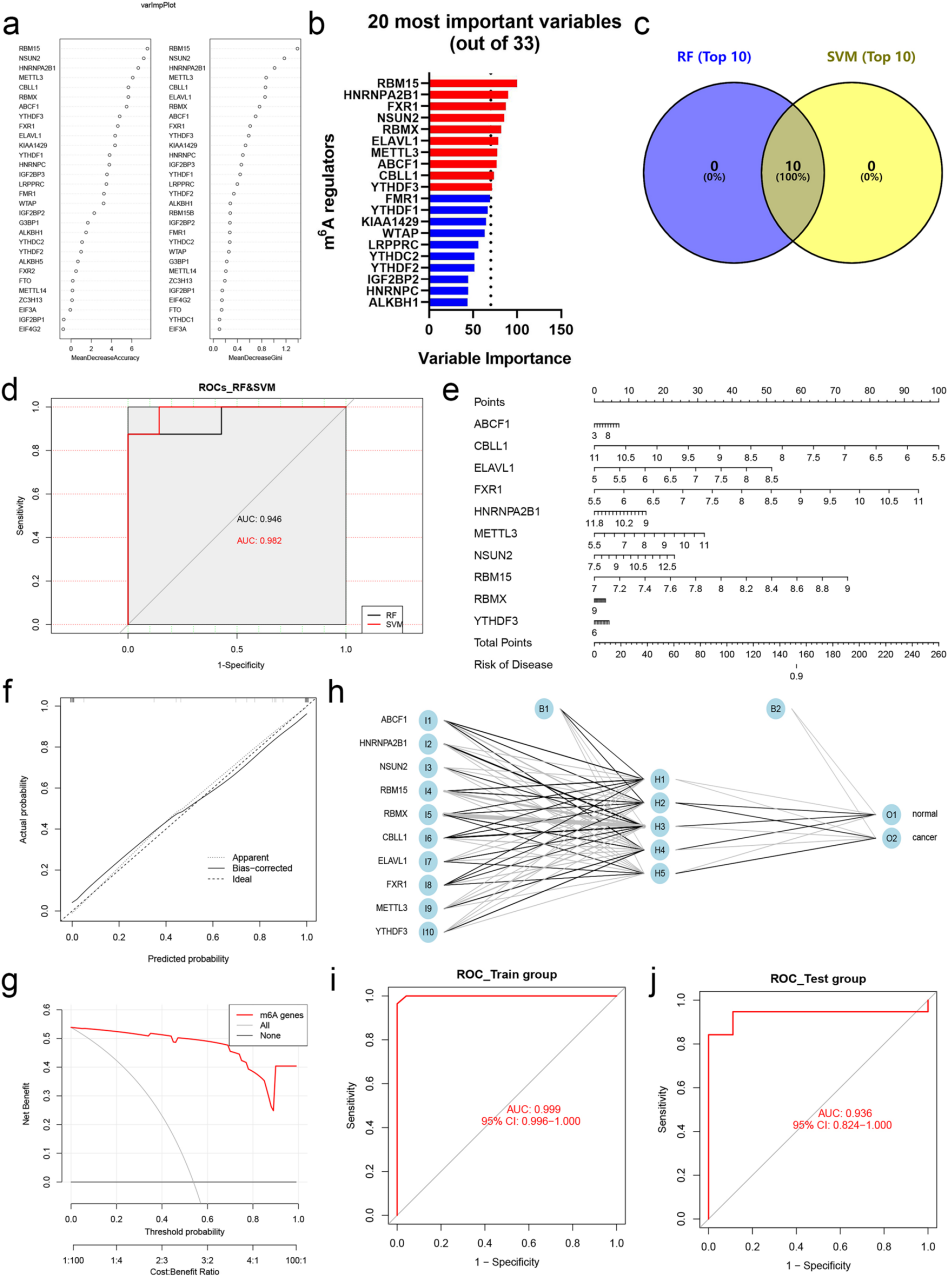
Diagnostic model construction and key m^6^A regulators identification. (**a**) Variable importance screening based on RF. (**b**) Variable importance screened via SVM. (**c**) Venn diagram showed the top 10 candidate regulators contained both in RF and SVM. (**d**) ROC curves based on machine learning methods for diagnostic probabilities. (**e**) The nomogram diagnostic prediction model based on ten filtered m^6^A regulators. (**f**) The calibration plots suggested the comparison between prediction and actual outcome for incidence probabilities in the nomogram model. (**g**) The decision curve analysis showed the net benefit in the nomogram model. (**h**) Establishment of CC diagnosis model with m^6^A factor as input layer based on ANN method. (**i**) ROC curves described the predictive ability of ANN model for CC incidence probabilities with the GSE63514 as train group. (**j**) ROC curves described the predictive ability of ANN model for CC incidence probabilities with the GSE6791 as test group.

To discriminate the potential functions of m^6^A regulators in CC, a diagnostic model was first constructed to provide a new viewpoint of CC diagnosis and prevention. Before commencing, 70% of patients in GSE63514 were used as the training set randomly, and the rest samples were defined as the validation set. The random forest (RF) model demonstrated that RBM15, NSUN2, HNRNPA2B1, METTL3, CBLL1, ELAVL1, RBMX, ABCF1, FXR1, and YTHDF3 were the top ten elements among all regulators (Fig. 2a, Fig. S2a,b). Moreover, RBM15, HNRNPA2B1, FXR1, NSUN2, RBMX, ELAVL1, METTL3, ABCF1, CBLL1 and YTHDF3 were identified by the support vector machine (SVM) model (Fig. 2b, Fig. S2c). Based on this, ten common regulators were recognized as key factors in CC diagnostic model (Fig. 2c, Fig. S2d). ROC curves of RF and SVM models showed high accuracy (AUC = 0.946 for RF model and AUC = 0.982 for SVM model; Fig. 2d). Moreover, a nomogram containing regulators and a calibration curve was performed with good accuracy for CC risk prediction (Fig. 2e,f). Decision curve analysis (DCA) was developed showing the benefit, as well as the AUC, were significantly improved (Fig. 2g).

Subsequently, to further probe the function of m^6^A regulators in CC diagnosis, an artificial neural network (ANN) was constructed via ten key elements mentioned above (Fig. 2h). For the training set, the ROC curve showed the ANN model had an extraordinary accuracy in CC diagnosis (AUC = 0.999; Fig. 2i), and the AUC of the test set (patients obtained from GSE6791) was 0.936 (Fig. 2j). These findings clarified that m^6^A regulators played an essential role in CC, which might provide a new perspective for the clinical diagnosis of CC.

### Prognostic value of m^6^A RNA methylation regulators and a risk signature constructed with significant ones

**Figure 3:**
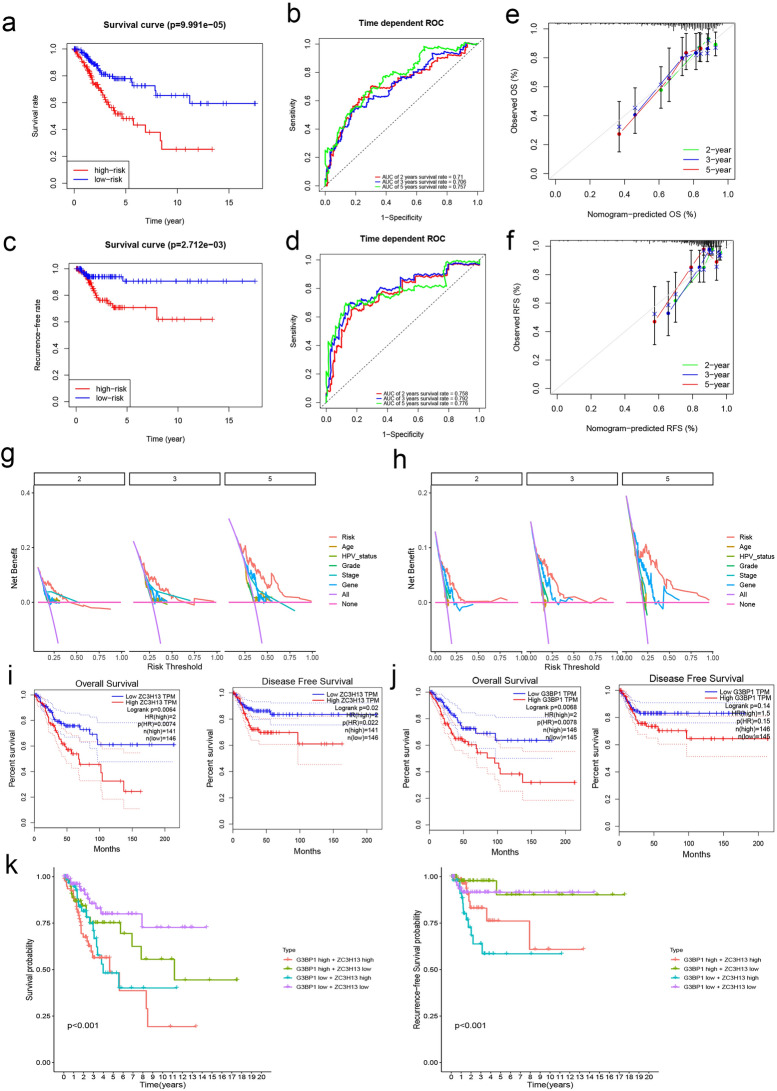
Prognostic model construction and key m^6^A regulators identification. (**a**) The Kaplan–Meier survival curves for CC patients with high- and low-risk. (**b**) ROC curves described the predictive ability of OS-LASSO model for 2-, 3-, and 5-year survival probabilities. (**c**) The Kaplan–Meier survival curves for CC patients with high- and low-risk. (**d**) ROC curves described the predictive ability of RFS-LASSO model for 2-, 3-, and 5-year survival probabilities. (**e,f**) The calibration plots suggested the comparison between prediction and actual outcome for 2-, 3-, and 5-year survival probabilities in the nomogram model for both OS (**e**) and RFS (**f**). (**g,h**) Decision curve analysis for the evaluation of the net benefits of riskscore, Age, HPV_Status, Grade, Genes (ZC3H13 expression) and Stage at 2-, 3-, and 5-year for both OS model (**g**) and RFS model (**h**). (**i**) KM analysis for patients with ZC3H13 different expression level for OS and RFS using GEPIA online tool. (**j**) KM analysis was performed in OS and RFS patients with different expression levels of G3BP1 using the GEPIA online tool. (**k**) Multivariate Kaplan–Meier survival curves for patients with different expression level of ZC3H13&G3BP1, which were selected by LASSO-Cox regression algorithm for OS and RFS prediction.

Furthermore, we attempted to explore the prognostic effects of regulators in CC. We developed the least absolute shrinkage and selection operator (LASSO) Cox regression algorithm using 33 m^6^A regulators for overall survival (OS) (Fig. S3a) and recurrence-free survival (RFS) (Fig. S3b) prediction in CC, respectively. For the OS prediction model, eight regulators (FMR1, G3BP1, HNRNPA2B1, LRPPRC, METTL16, WTAP, YTHDF3, and ZC3H13) were identified, and seven factors (YTHDF1, FXR2, YTHDC2, G3BP1, IGF2BP1, RBMX, and ZC3H13) were filtered, independently. The risk score of OS model for each patient was calculated with the following formula: Riskscore = 0.014 × ZC3H13 + 0.008 × YTHDF3 + 0.007 × WTAP + 0.001 × LRPPRC + 0.008 × HNRNPA2B1 + 0.004 × G3BP1 − 0.016 × FMR1 − 0.003 × METTL16. And similarly, risk score of RFS model was calculated with the following formula: Riskscore = 0.031 × ZC3H13 + 0.011 × RBMX + 0.004 × IGF2BP1 − 0.003 × G3BP1 − 0.007 × YTHDC2 − 0.010 × FXR2 − 0.018 × YTHDF1. Afterwards, the Kaplan–Meier survival curve results confirmed that the risk signature had significant predictive power in OS prediction (*P* < 0.001; Fig. 3a). Similarly, consistent results were found in RFS analyses (*P* = 0.003; Fig. 3c). Finally, the evaluation of the LASSO regression model using the receiver operating characteristic (ROC) curves and area under the curve (AUC), and results revealed that the signature had more accurate prognostic predictability in the training set (AUC_OS_ = 0.757 for 5-year survival rate and AUC_RFS_ = 0.776 for 5-year recurrence-free rate; Fig. 3b,d) for CC prognosis prediction. Interestingly, our single-gene ROC analysis demonstrated that ZC3H13 had the highest AUC in both OS (AUC = 0.698; Fig. S3a) and RFS (AUC = 0.711; Fig. S3b) predictions, which indicated the potential role of ZC3H13 in patient prognosis. We then constructed a nomogram, which included the clinicopathological characteristics with ZC3H13 expression of patients, and evaluated the accuracy of the model through the calibration curve (Fig. S3c,d). An obvious trend was notable that the models had a better prognosis prediction value (Fig. 3e,f). Subsequently, DCA plots illuminated that the risk score obtained from LASSO got the highest net benefit than other clinical-pathological features for both OS (Fig. 3g) and RFS (Fig. 3h). The patients with high-risk scores exhibited reduced OS and RFS (Fig. S3e,f).

To expound on the scientificity and stability of predictive models, internal or external validation of OS and RFS models were also performed. Principal component analysis (PCA) as well as t-distributed stochastic neighbor embedding (t-SNE) processes were performed to show the patients with a different risk score based on prognostic models, and the outcome suggested that risk scores differentiated patients sufficiently (Fig. S4a,b). Moreover, several gynecological cancers contain Breast invasive carcinoma (BRCA), Ovarian serous cystadenocarcinoma (OV), Uterine Corpus Endometrial Carcinoma (UCEC), and an HPV-associated tumor, Head and Neck squamous cell carcinoma (HNSC) were selected for OS prediction model validation. In addition, the GSE44001 cohort was acquired to verify the RFS model. The consequence indicated that the prognostic model conducted could convincingly define the survival risk or recurrence-free survival risk with reasonable accuracy (*P*s < 0.001, ROCs > 0.6; Fig. S4c,d).

Risk scores based on m^6^A regulators and the LASSO model were sufficient to forecast patient prognostic risk robustly, but several interesting factors that combined into risk scores attracted our attention. ZC3H13 and B3BP1 were common variables in both OS and RFS models, although their direction of effect on prognostic outcomes appeared to be inconsistent. Subsequently, the KM plots downloaded from GEPIA revealed that only upregulation of ZC3H13 expression was significantly associated with worse prognosis (*P* = 0.006 for OS and *P* = 0.002 for RFS; Fig. 3i), but not G3BP1 (*P* = 0.007 for OS and *P* = 0.140 for RFS; Fig. 3j). When the bivariate K-M analysis was carried out, we found that the presence of ZC3H13 significantly predicted patient outcomes (*P*s < 0.01, Fig. 3k), patients with low expression of both had better prognosis, while patients with high expression of both had worse prognosis. It was evident that this trend was highly correlated with the expression level of ZC3H13. Briefly, ZC3H13 was a vital factor in CC prognosis prediction, and combined with G3BP1, can improve the predictive power.

**Figure 4:**
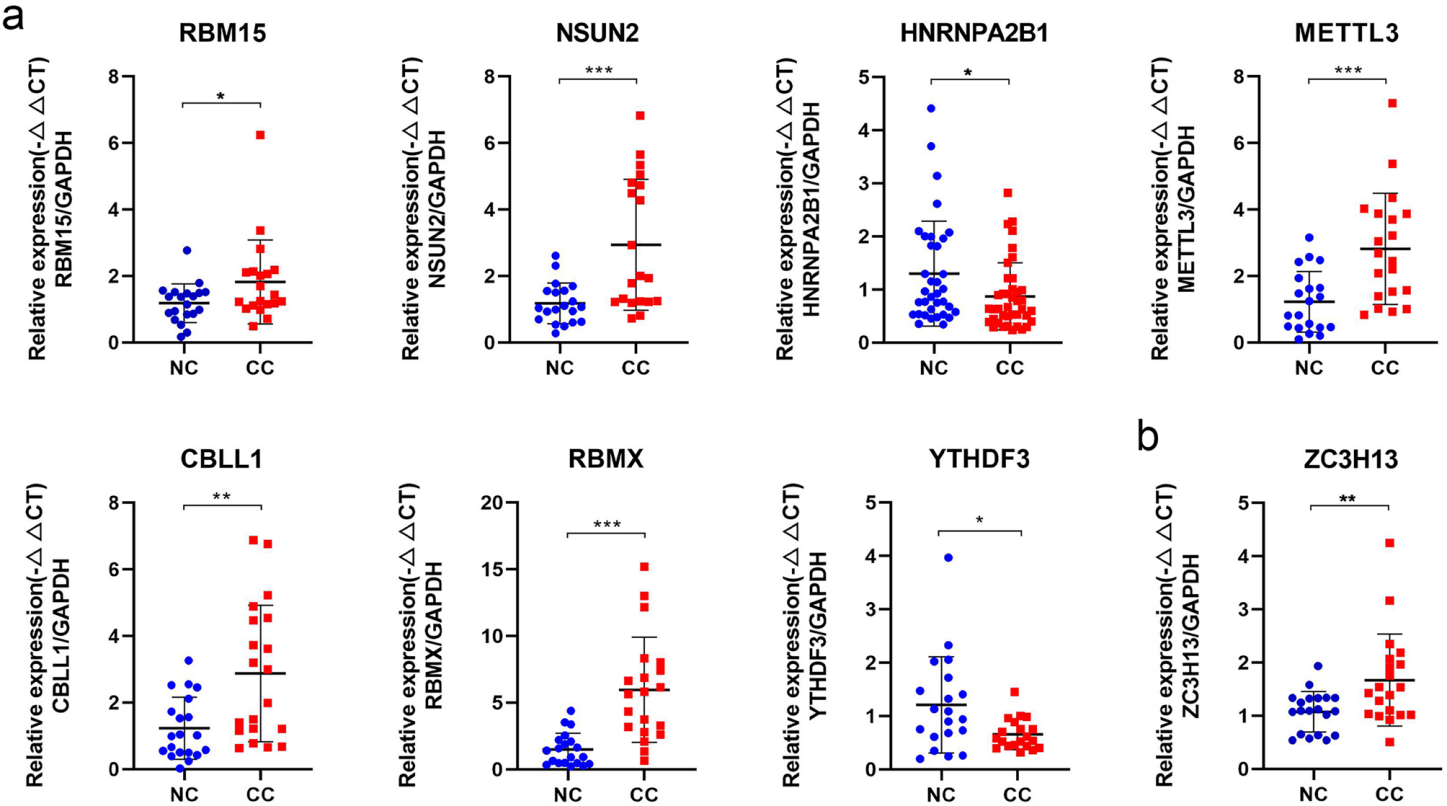
RNA expression detection of 20 pairs human cervical tissues using Real-time Quantitative PCR Detecting System. The expression comparison of RBM15, NSUN2, HNRNPA2B1, METTL3, CBLL1, RBMX, YTHDF3, and ZC3H13.

Considering whether key factors could be used as stable CC biomarkers, we detected seven diagnostic elements and one prognostic factor in 20 pairs of population tissues. The comparison elucidated that RBM15 (*P* = 0.046; Fig. 4a), NSUN2 (*P* = 0.001), METTL3 (*P* = 0.001), CBLL1 (*P* = 0.003), RBMX (*P* < 0.001), and ZC3H13 (*P* = 0.008; Fig. 4b) were significantly up-regulated in CC tissues, while the expressions of HNRNPAB1 (*P* = 0.027) and YTHDF3 (*P* = 0.033) and showed the opposite trend.

### Two CC subgroups were identified by consensus clustering and immune-associated exploration based on m^6^A RNA methylation regulators

**Figure 5:**
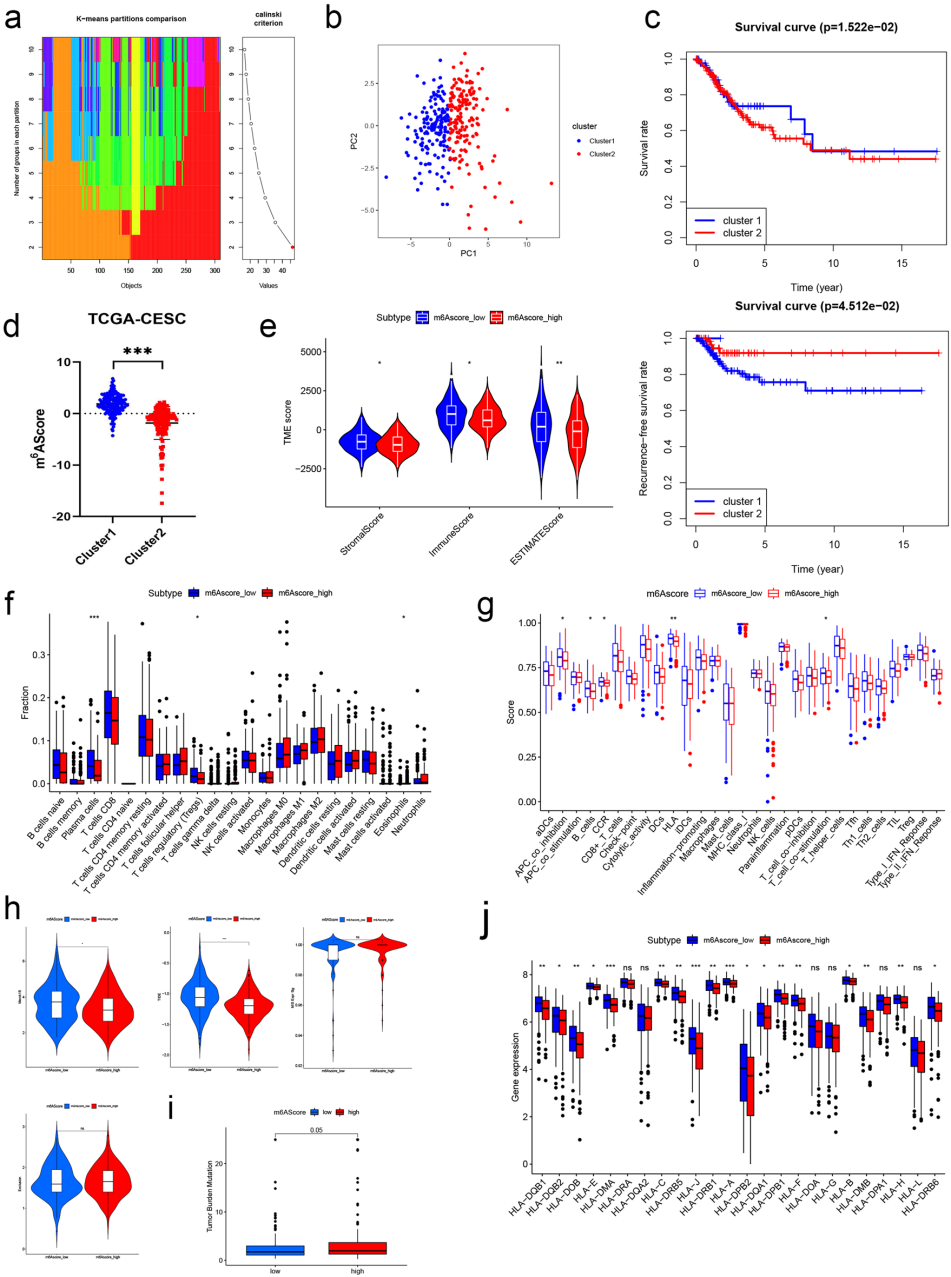
Identification and characteristic description of consensus clustering. (**a,b**) Consensus clustering for k = 2 based on m^6^A regulators (**a**) and visualization by PCA plot (**b**). (**c**) Kaplan–Meier survival curves for patients in different subgroups, OS and RFS. (**d**) Representation of the groups character by m^6^AScore. (**e**) Analysis and comparison of tumor microenvironment in patients with different m^6^AScore level. (**f**) The comparison of proportion of 22 immune cells in 309 patients of different m^6^AScore group. (**g**) Immune function analysis and comparison in patients with different m^6^AScore group. (**h**) Immune escape analysis (Merck18, TIDE, MSI score and T cell Exclusion) and comparison in patients with different m^6^AScore level. (**i**) Tumor mutation burden between different m^6^AScore and TMB. (**j**) Box plot showed the comparison of HLA family genes expression between different m^6^AScore groups. *P < 0.05; **P < 0.01; ***P < 0.001; ****P < 0.0001.

To further explore the effect of m^6^A RNA modification in CC, we calculated cluster fitting values of k = 2 to 10 on 306 cancer samples based on the expression correlation of 33 regulators. The results, as shown in Fig. 5a and Fig. S5a, indicated that k = 2 was relatively optimal for further analysis. Based on this, the cluster 1 (n = 151) and cluster 2 (n = 155), respectively. Furthermore, to intuitively obtain the effect of two subgroups and reflect the reliability of our results, we calculated PCA and t-SNE analysis based on two subgroups. We found the clustering results could effectively distinguish the two clusters (Fig. 5b, Fig. S5b). Subsequent KM analysis results indicated that cases in cluster 1 had better prognostic status than in cluster 2 (*P* = 0.015 for OS and *P* = 0.045 for RFS; Fig. 5c). Gene Set Enrichment Analysis (GSEA) was either conducted to investigate the enrichment of the genes in two clusters. The result showed that mitochondrial drug metabolism (P450 and other enzymes) was significantly enriched in cluster 1, while cell cycle, DNA replication, nucleotide excision repair, and spliceosome-associated biological processes was found in cluster 2 (Fig. S5c).

To clarify the association between m^6^A regulators and clusters, an m^6^A-related score, named m^6^AScore, based on 33 key factors was calculated via PCA analysis to quantitatively describe the m^6^A level of each patient. Obviously, patients in cluster 1 had higher m^6^AScore than patients in cluster 2 (*P* < 0.001; Fig. 5d). After KM survival analysis, a trend was revealed that patients with higher m^6^AScore had better prognostic status (*P* = 0.003 for OS and *P* = 0.005 for RFS; Fig. S5d,e). Understandably, most of the 33 genes were significantly different expression in two clusters (Fig. S5f) and m^6^AScore groups (Fig. S5g) based on m^6^A regulators. These results suggested that m^6^AScore based on m^6^A regulators could also predict the prognostic risk of patients.

Few studies have delved into the association of m^6^A regulators and immune function comprehensively. Firstly, stromal score, immune score, and tumor purity analysis were developed via the ESTIMATE algorithm, and the differentiation of patients in different m^6^AScore groups was identified. The result showed that StromalScore, ImmuneScore, and total score, ESTIMATEScore, were significantly lower in the high-m^6^AScore group than in the low-m^6^AScore group (*P*s < 0.05; Fig. 5e). Subsequently, we performed immune infiltration analysis using the same cases mentioned above and explored the association between different m^6^AScore groups (Fig. 5f). However, only the abundance of plasma cells, regulatory T cells (Treg), and eosinophils had obviously difference between low- and high-m^6^AScore patients. Analysis of immune function were executed subsequently and results implied that co-inhibition of antigen presenting cells (APCs), B cells, Chemokine receptors (CCRs), HLA, and T cell co-stimulation in high-m^6^AScore group were less than low-m^6^AScore group (*P*s < 0.05; Fig. 5g). Hereafter, TIDE scores reflecting the patient sensitivity to immune checkpoint inhibitors (ICIs) were calculated to explore the discrepancy between high- and low-m^6^AScore groups. As Fig. 5h showed, Merck18 and TIDE score were reduced in high-m^6^AScore group, but MSI score and T cell Exclusion showed no significant difference. The tumor mutation burden (TMB) in different m^6^AScore groups were developed and an insignificant difference was observed (*P* = 0.050, Fig. 5i). The following KM curves proved that patients with high TMB level had better RFS (*P* < 0.001; Fig. 5h), but not OS (*P* = 0.152). Only when m^6^AScore and TMB were analyzed together, was it observed that the group with high-TMB+ low-m^6^AScore had the best prognostic status (*P* = 0.015 for OS and *P* < 0.001 for RFS; Fig. 5i). In general, the results of this part showed that m^6^AScore was significantly associated with tumor microenvironment, immune infiltration, immune function, immune escape, and TMB in CC patients. M^6^AScore could be used as an indicator of patients' immune status, immune escape and prognosis, but its role limited.

**Figure 6:**
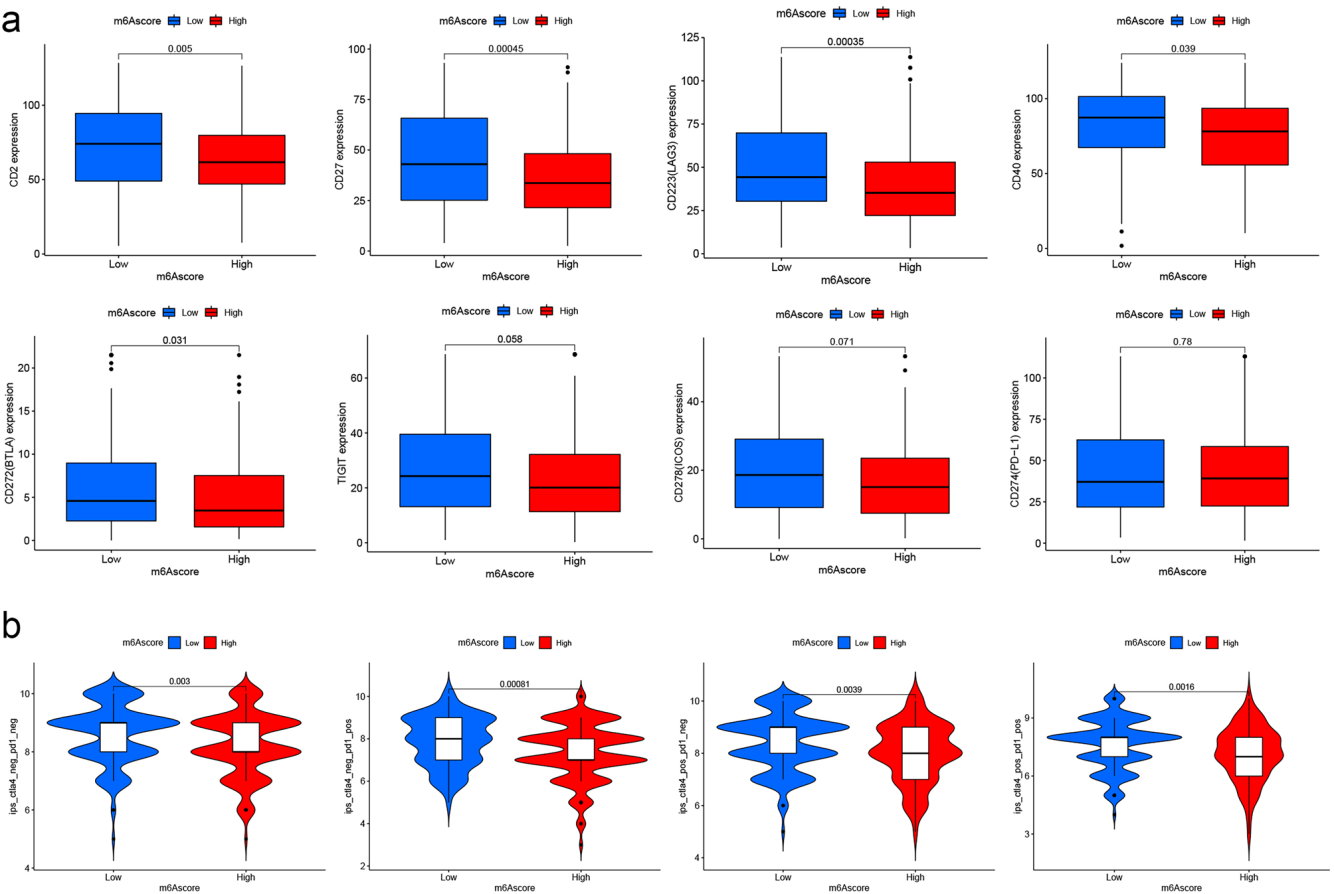
Immune checkpoint expression analysis and immunotherapy exploration based on M^6^AScore. (**a**) Expression differences in immune checkpoints (CD2, CD27, LAG3, BTLA, TIGIT, ICOS) between different m^6^AScore groups. (**b**) The comparison of the relative distribution of immunophenoscore (IPS) between different m^6^AScore groups.

To precisely connect m^6^AScore and immune process, we shifted our focus to the HLA family mentioned above and a comparison between different m^6^AScore groups was performed. Unexpectedly, 18 of the 24 traits had lower levels in the high-m^6^AScore group, and other insignificant traits showed the same downward trend (Fig. 5j). This motivated us to explore the association between m^6^AScore and immune checkpoint expression. A total of 15 immune checkpoints (BTLA, CD2, CD200R, CD244, CD27, PD-L1, CD28, CD40, CD80, ICOS, KLRC1, KLRD1, LAG3, SIRPA, and TIGIT) were identified for subsequent analysis. The results clarified that CD2, CD27, LAG3, CD40, and BTLA had less abundance in high-m^6^AScore groups (*P*s < 0.05; Fig. 6a). No distinct differences were observed in the expression abundances of TIGIT, ICOS, PD-L1, and others in between different m^6^AScore groups. At the end of this section, to predict the response of ICIs, we determine the association in m^6^AScore and immunophenoscore (IPS) in CC patients. As Fig. 6b illustrated, patients with low-m^6^AScore had higher PD-1 and CTLA4/PD1 scores (*P*s < 0.05), elucidating patients with the high immunogenicity on ICIs. This was consistent with the result of lower expression of immune checkpoints in high-m^6^AScore group mentioned above. In conclusion, there were significant differences in m^6^AScores between immunotherapy non-responders and responders, and m^6^AScore could provide a new reference for individualized treatment of CC patients.

### Drug sensitivity analysis for clinical chemotherapy strategies

**Figure 7:**
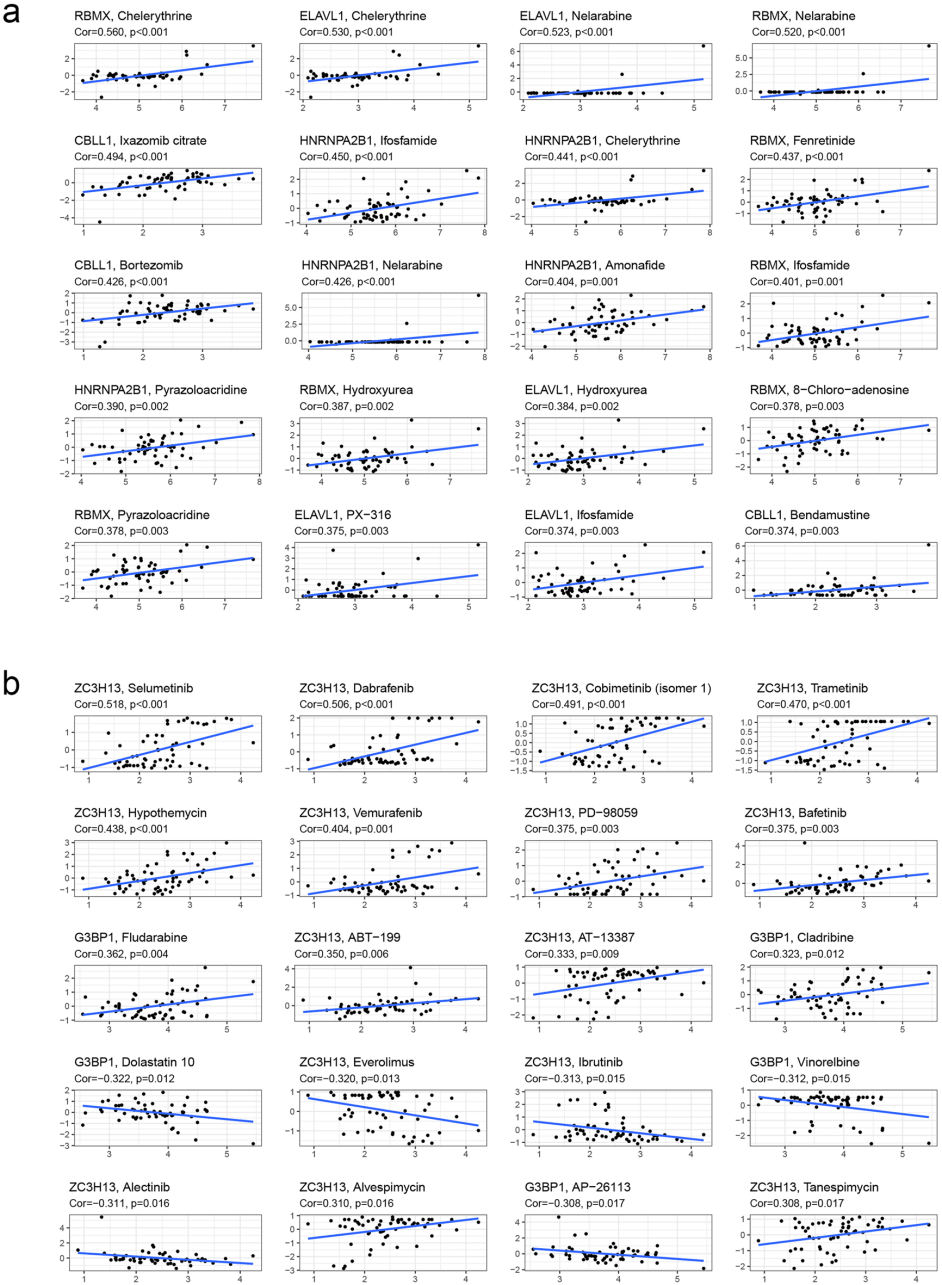
Analysis of treatment strategies for diagnosis and prognosis based on m^6^A regulators. (**a**) The scatter diagram showed the association between the drugs and key regulators, which might provide new clues to uncover potential mechanisms for CC diagnosis. (**b**) The association between the drugs and key prognostic regulators.

It was mentioned earlier that there were ten key molecules (RBM15, NSUN2, HNRNPA2B1, METTL3, CBLL1, ELAVL1, RBMX, ABCF1, FXR1, and YTHDF3) in the CC diagnostic model constructed and similarly two key elements (ZC3H13 and G3BP1) in the prognostic model (Figs. 2, 3). Based on this, we sought to explore the association of key factors with widely recognized chemotherapeutic agents. Figure 7 revealed that RBMX was strong positive correlation with Chelerythrine (R = 0.560, *P* < 0.001), Nelarabine (R = 0.520, *P* < 0.001), and Fenretinide (R = 0.437, *P* < 0.001); ELAVL1 was identified obvious related to Chelerythrine (R = 0.530, *P* < 0.001), Nelarabine (R = 0.523, *P* < 0.001), and Hydroxyurea (R = 0.384, *P* = 0.002). When the perspective turns to the prognostic factors, the results suggested that Selumetinib (R = 0.518, *P* < 0.001), Dabrafenib (R = 0.506, *P* < 0.001), and Cobimetinib (R = 0.491, *P* < 0.001) were filtered as the potential ZC3H13 associated drugs. As Fig. 7 showed, Chelerythrine, Nelarabine, Ifosfamide, and Selumetinib were considered as potential chemotherapeutic agents to target these factors.

In addition, we also obtained drug data through Connectivity Map (cMAP) database and performed association analysis with m^6^AScore to explore the clinical treatment strategies for CC patients. Comparison consequence were demonstrated that patients with low-m^6^AScore marked sensitivity to chemotherapeutic agents, including AKT inhibitor VIII (*P* < 0.001; Fig. S7a), BIRB.0796 (a p38 MAPK inhibitor; *P* < 0.001) and FH535 (a Wnt/β-catenin inhibitor; *P* < 0.001), but NVP.TAE684 (an ALK inhibitor; *P* < 0.001) were opposite to them. Cisplatin, Paclitaxel, and Gemcitabine, which are commonly used clinical chemotherapy drugs for malignant tumors, also had significant differences in the effects of different m^6^AScore patients. CC patients from low-m^6^AScore group were more sensitive to Cisplatin and Gemcitabine, but not to Paclitaxel. Our results demonstrated that the m^6^AScore calculated based on 33 m^6^A regulators can be used to predict the sensitivity of patients to chemotherapy drugs, which might be of great significance for clinical chemotherapy drugs.

### Correlational exploration of m^6^A RNA modification with cell cycle and programmed death

**Figure 8:**
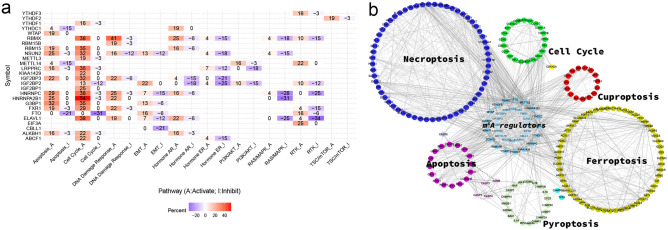
Correlation analysis of m6A regulators with cell cycle and programmed death. (**a**) The essential pathway enrichment analyses of 33 m^6^A regulators by GSCA. (**b**) The correlation network between m^6^A regulators and programmed death genes of interest, which including cell cycle, apoptosis, pyroptosis, necroptosis, ferroptosis and cuproptosis (|R|> 0.3 and *P* < 0.05). Solid lines represent positive correlations, dashed lines represent negative correlations. The thicker the line, the stronger the correlation between the two edges.

Considering the potential molecular mechanism of m^6^A regulators in CC, a functional enrichment analysis was developed. By Gene Set Cancer Analysis (GSCA) database, the active of cell cycle and apoptosis were the significantly acquired pathways (Fig. 8a). Correlation analysis was subsequently fulfilled to probe potential associations of m^6^A regulators with gene lists of cell cycle and five programmed cell death, which included ferroptosis, pyroptosis, apoptosis, necroptosis, and cuproptosis. Figure 8b revealed a network of molecular with extensive connection (Rs > 0.300; *P* < 0.050). In summary, results exhibited cell cycle and cell programmed death were closely related to m^6^A regulators, which were the essential pathways for CC progression.

## Discussion

In recent years, epigenetic modification has been widely studied. Existing evidence shows that epigenetic modification exists in various molecular biological processes, and it has a significant role in the occurrence and development of cancer^27–31^. As the more critical one, m^6^A RNA modification has also been shown to have an important direct relationship with cancers^32–36^. In this study, we demonstrated that the expression of m^6^A RNA modification regulators in CC was closely related to its diagnosis and prognosis. The functional enrichment results revealed the feasible key signaling pathways of m^6^A modification in CC, including cell cycle and cell programmed death. Next, the m^6^AScore was calculated via PCA algorithm and used to investigate the distinction between CC patients from different clusters obtained by consensus clustering. Fortunately, it was found that the lower the m^6^AScore, the better the prognosis of patients. Immune characterization and tumor microenvironment analysis subsequently showed significant differences in patients with low- or high-m^6^AScore, suggesting a potential association between m^6^A modification and immune processes. It is highly consistent with known reports^37–39^.

In the present study, we explored the diagnostic value and identified ten key regulators including RBM15, NSUN2, HNRNPA2B1, METTL3, YTHDF3, FXR1, RBMX, ELAVL1, CBLL1, and ABCF1, using RF and SVM models. Among them, RBM15 was the most crucial. Subsequent ANN results suggested that our model was accurate in both training and validation cohorts (Fig. 2). Although the current literature on m^6^A is numerous^19,40^, there are few reports identifying RBM15 as an essential biomarker for CC prognostic. The study found that RBM15 could increase the m^6^A level of TMBIM6 mRNA, and increase its stability after recognition by the reader protein, promoting the malignant progression of laryngeal squamous cell carcinoma^41^. Based on this, a hypothesis was proposed that RBM15 also has a non-negligible potential role in the occurrence and development of CC by mediating the m^6^A level of targets. After experimental verification (Fig. 4b), RBM15 was indeed significantly up-regulated in patients, but its in-depth molecular mechanism in CC was the focus of our future work. HNRNPA2B1 was another important factor, which was identified as an oncogene in head and neck cancer and could promote Akt/PKB signaling by upregulating the RONΔ165 isoform, thereby promoting epithelial-mesenchymal transition of head and neck cancer cells^42^. HNRNPA2B1 increased the stabilization of ILF3 mRNA through m^6^A modification, which in turn increased AKT3 expression to promote multiple myeloma progression^43^. Although the qPCR assay found that HNRNPA2B1 was significantly down-regulated in CC patients, which was inconsistent with literature reports and the result obtained from datasets, including TCGA-CESC, GSE63514, and GSE6791. It was consistent with them that HNRNPA2B1 was served as a risk factor in OS prognostic models, implying its complex mechanism in CC. Meanwhile, we also considered to detect the relative expression of HNRNPA2B1 again after expanding the tissue sample size.

We distinguished an eight-m^6^A RNA modification gene signature containing FMR1, G3BP1, HNRNPA2B1, LRPPRC, METTL16, WTAP, YTHDF3, and ZC3H13 for CC OS prediction. Similarly, seven regulators (FXR2, G3BP1, IGF2BP1, RBMX, YTHDC2, YTHDF1, and ZC3H13) were identified for RFS prediction. According to the risk obtained by the LASSO Cox model, we found that this score accurately distinguished patients with different prognostic risks. ZC3H13 and G3BP1 were served as common indicators for both OS and RFS prediction. ZC3H13 could be selected alone for CC prognosis prediction, and the ROC curve showed that its AUC is larger than other members and close to the total AUC of the model (Fig. S3a,b). We next attempted to mix both of them to predict CC prognostic risk. The KM plots suggested that the predictive risk was significant when ZC3H13 and G3BP1 were combined. For ZC3H13, studies have identified it as an important prognostic predictor in Glioblastoma^44^ and CC^15^. It is worth mentioning that our results suggested that ZC3H13 was significantly up-regulated in CC tissues (Fig. 4b), which was consistent with the reported trend^15^, indicating that ZC3H13 was a stable biomarker.

Consensus clustering analysis based on m^6^A regulators was executed to divide CC patients into two clusters with m^6^AScore. Patients in cluster 1 had higher m^6^AScores and worse prognosis, whereas patients in cluster 2 had the exact opposite. In recent years, it has been reported in the literature that m^6^A regulators can participate in regulating the occurrence, development, and treatment of various tumors by affecting immune-related processes, including but not limited to immune response^45,46^, immune checkpoint expression^47,48^, and immune escape^49,50^. Immune cell infiltration analysis and tumor microenvironment analysis clarified that the immune status and microenvironment were significantly different in m^6^AScore groups. The level of immune cell infiltration, immune TIDE, and TME score in the patients from high-m^6^AScore group were clear lower than those in patients with low-m^6^AScore. In subsequent analysis of immune infiltration, immune escape and TMB, the results showed that m^6^AScore correlated significantly with these features, but very limited. We speculated that the m^6^AScore constructed based on the abundance of m^6^A regulators did not show a strong correlation with the immune infiltration and immune escape status of patients, but the m^6^A-regulated target genes were directly related to the immune process^51–53^. Therefore, the m^6^AScore showed a weak correlation with immune status. In addition, the information loss caused by dimensionality reduction during the construction of m^6^AScore may also weaken the association to a certain extent. Although no significant differences were observed in TMB and MSI in different m^6^AScore groups, we found that the HLA and immune checkpoint expressions of patients with low-m^6^AScore were significantly lower than those in patients with high-m^6^AScore. Immunotherapy analysis also found that patients with low-m^6^AScore had better treatment benefits. For such patients, immunotherapy is a scientifically effective protective measure. For patients with high-m^6^AScore, immunotherapy might not be a high-benefit approach, possibly due to the high proportion of patients with advanced cancer. The pattern of low expression of immune checkpoints in patients with worse prognosis was previously reported in studies^54^, which was consistent with our results. As for patients with high-m^6^AScore, NVP.TAE684 and Paclitaxel were more suitable for them.

There have been many literatures on the m^6^A molecules in CC, some of which are similar but not the same. Pan’s study^15^ analyzed 13 m^6^A regulators in the TCGA-CESC dataset and identified ZC3H13, YTHDF1, and YTHDC1 as OS-related factors. Consistently, we also identified ZC3H13 as a key factor on patient OS prediction, and both of our studies found ZC3H13 to be the most essential influencing factor (observed from the coefficients). Neither YTHDF1 nor YTHDC1 were in our model, and we presume the reason for the discrepancy may be the difference in the number of included independent variables, which would result in non-essential variables not being stably retained in the model. Furthermore, the expression trend of METTL3 was inconsistent with our experimental results. In addition to individual differences in the population, it was our conjecture that the small sample size causes biased results. In addition, complex mechanisms between RNAs and proteins may also lead to different outcomes. Zhang’s research^16^ explored the expression patterns of m^6^A molecules in CC and comprehensively analyzed the connection with immune-related processes. Although expression patterns were explored for both, we ultimately constructed m^6^AScore based on m^6^A regulator expression values rather than differential genes. This was the main difference between our two studies and the main reason for the difference in the results that follow. Most importantly, this study also constructed a cervical cancer diagnostic model and RFS prognostic model, and proposed chemotherapy regimens for the identified key targets, while complementing the shortcomings of immunotherapy in the study.

The limitations of this study should be considered when interpreting the results. The dataset we used when building the diagnostic model was GSE63514, and the validation dataset was GSE6791. However, most of this study is based on the TCGA database, which is a completely different group. The reason was that in the diagnostic model, we needed the population of different groups in the data to be as balanced as possible, and TCGA was difficult to meet this requirement (306 CC patients and 3 normal samples). In addition, the necessary survival data in the prognostic model was also difficult to obtain in the GEO dataset; there is not yet a public database that can simultaneously meet the requirements of both. We are currently constructing a balanced CC follow-up cohort and hope to fill this gap in future studies. Another important point is that the experiments on the expression of key m^6^A regulators in the study only did qPCR, and there was a lack of evidence from a large number of immunochemistry results. This deficiency will also be improved and published in the future work.

## Conclusion

In conclusion, our findings supported a systematic analysis that m^6^A regulators executed vital functions in the diagnosis, prognosis, immune microenvironment, and treatment of CC. And these mechanisms not yet completely elucidated today might be achieved by immune biological process, cell cycle, and cell programmed death. This study also offered a theoretical basis for CC clinical treatment.

## Supplementary Information

Supplementary Information.

## Data Availability

The datasets generated and analyzed during the current study are available in the TCGA (https://portal.gdc.cancer.gov/) and GEO (https://www.ncbi.nlm.nih.gov/geo/query/acc.cgi?acc=GSE63514; https://www.ncbi.nlm.nih.gov/geo/query/acc.cgi?acc=GSE6791) database. The other data, algorithms and code that support the findings of this study are available on request from the corresponding author. The algorithms and code were not publicly available due to privacy or ethical restrictions.
