# Supplementary material for: Gene signature of m6A RNA regulators in diagnosis, prognosis, treatment, and immune microenvironment for cervical cancer
Source: Sci Rep. 2022 Oct 21;12:17667. doi: 10.1038/s41598-022-22211-2 (PMC9587246; doi:10.1038/s41598-022-22211-2)
Supplement: Supplementary file 1 — Supplementary Information. [file 41598_2022_22211_MOESM1_ESM.pdf]

Supplementary Materials

1 Supplementary Figures:

Fig.S1

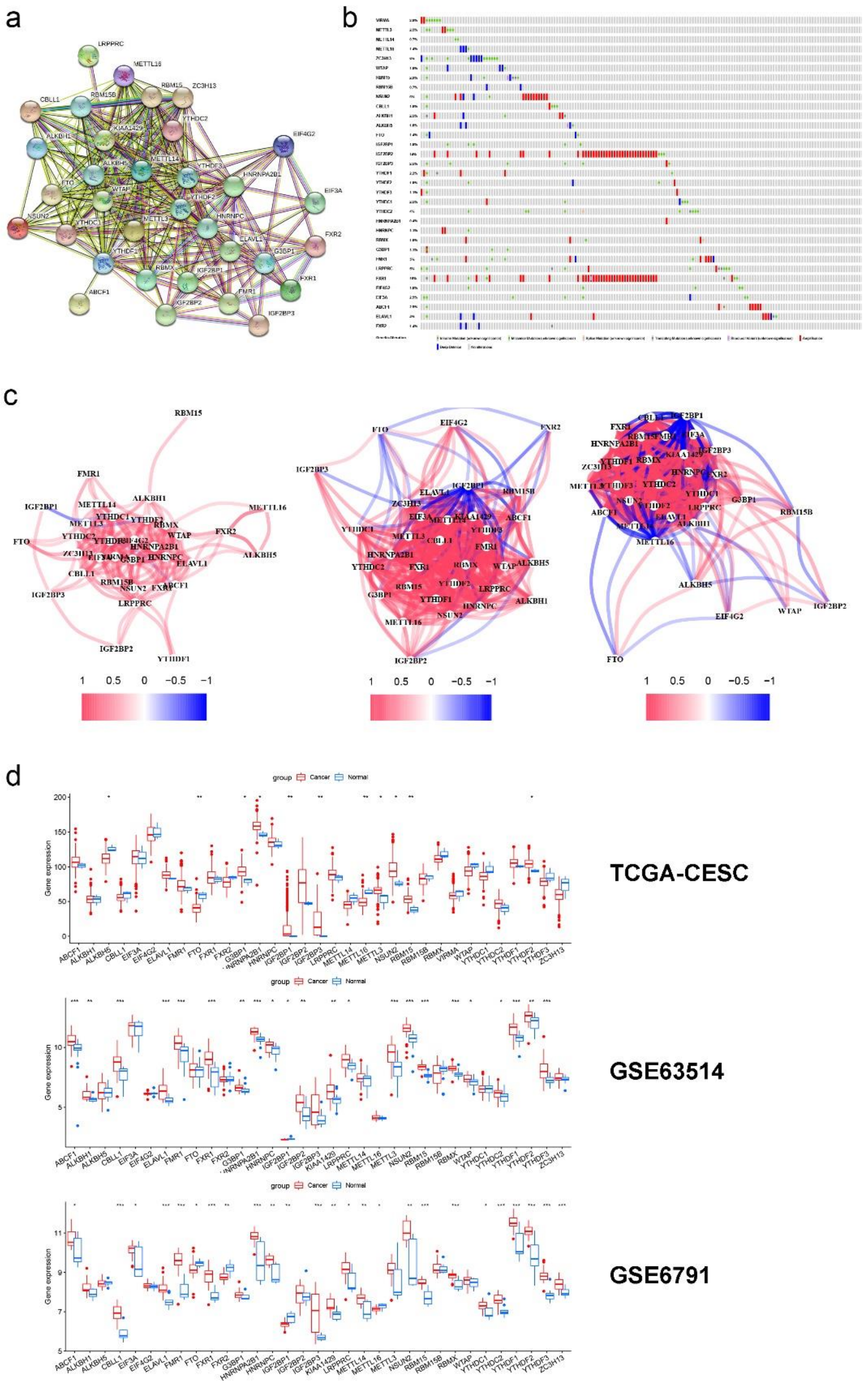

Fig.S2

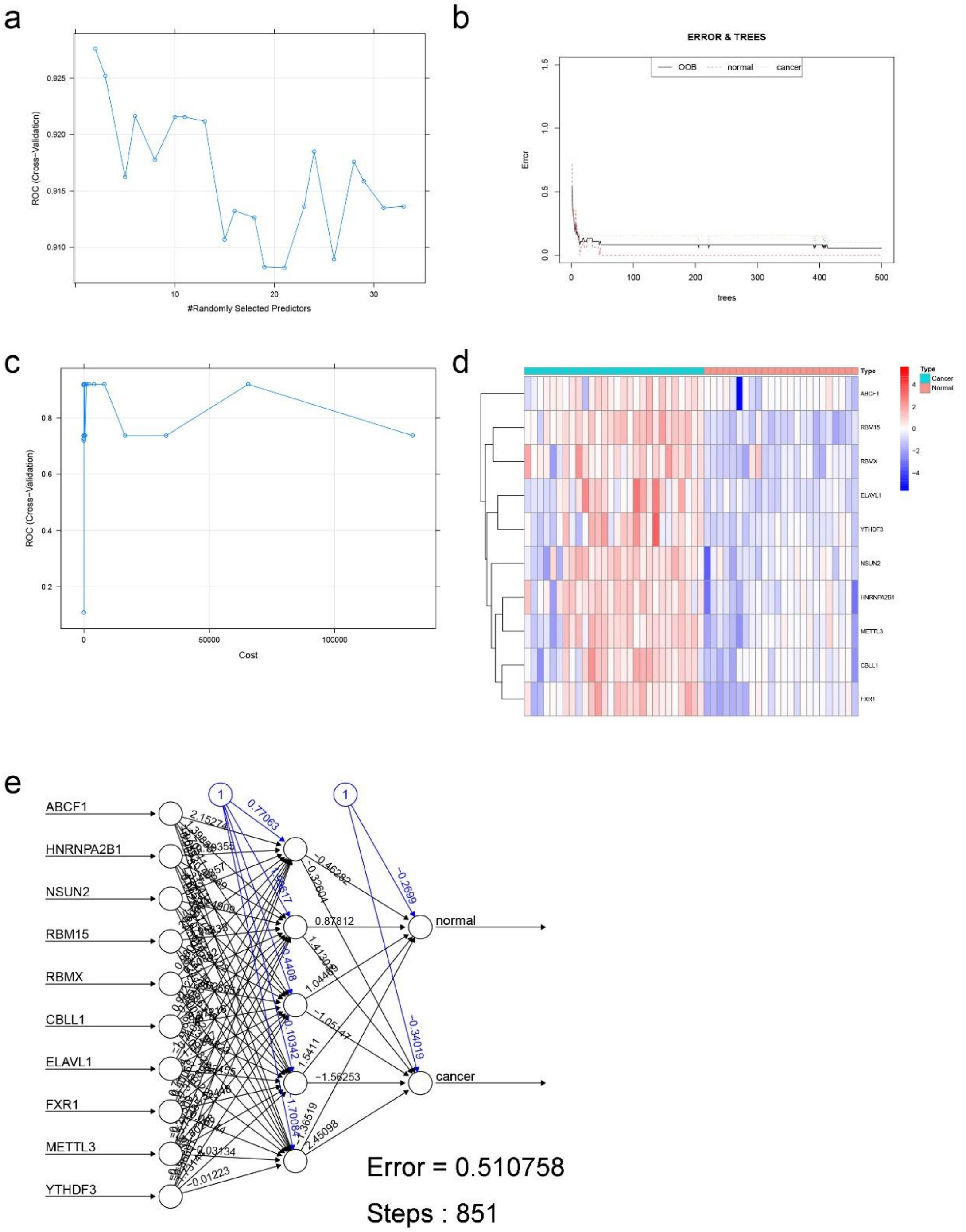
a
ROC (Cross-Validation)
#Randomly Selected Predictors
b
ERROR & TREES
OOB
normal
cancer
Error
trees
c
ROC (Cross-Validation)
Cost
d
Type
Cancer
Normal
ABCF1
RBM15
RBMX
ELAVL1
YTHDF3
NSUN2
HNRNPA2B1
METTL3
CBLL1
FXR1
e
ABCF1
HNRNPA2B1
NSUN2
RBM15
RBMX
CBLL1
ELAVL1
FXR1
METTL3
YTHDF3
normal
cancer
Error = 0.510758
Steps : 851

Fig.S3

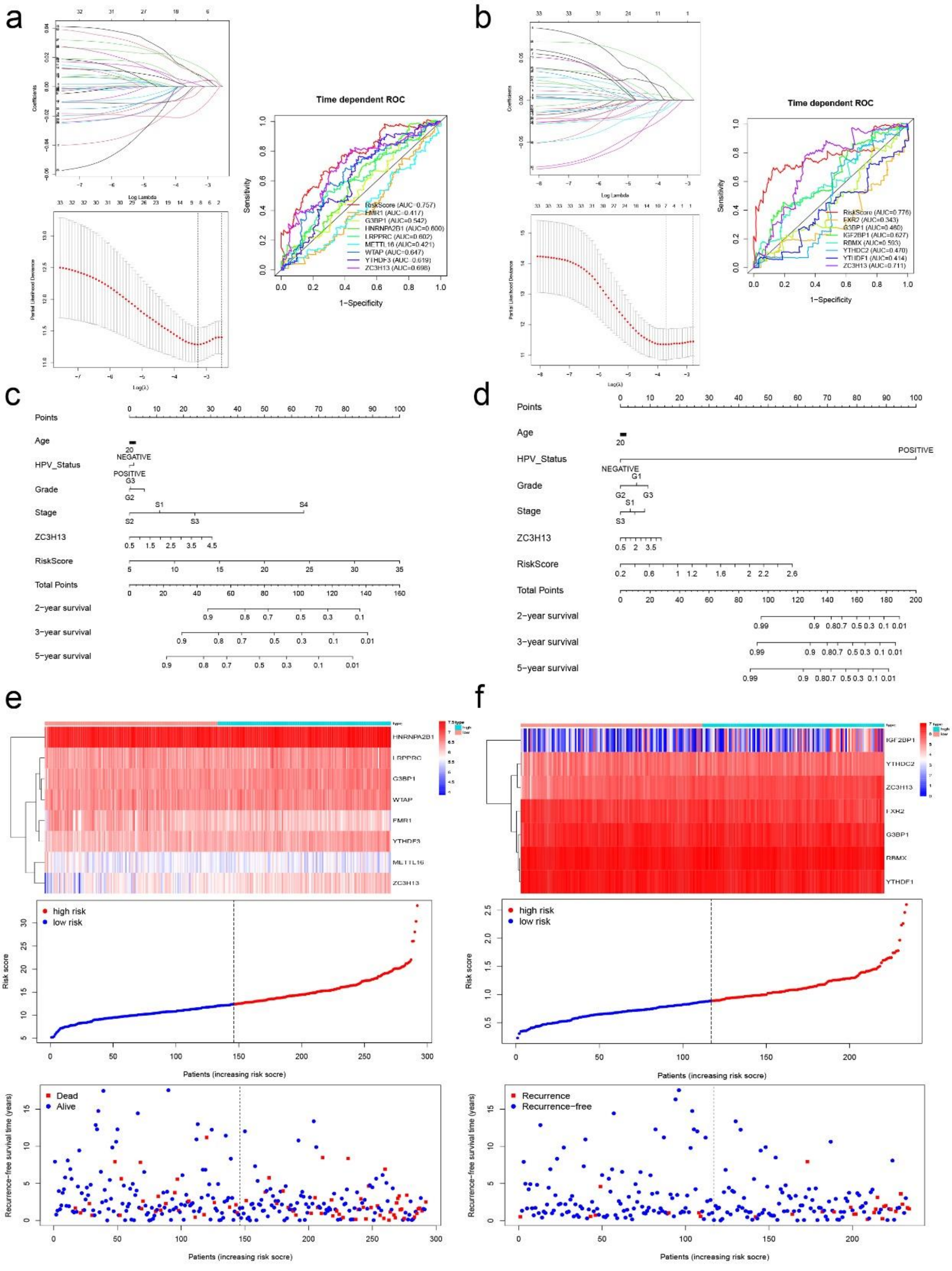
a
b
Time dependent ROC
Sensitivity
1−Specificity
RiskScore (AUC=0.757)
FMR1 (AUC=0.417)
G3BP1 (AUC=0.542)
HNRNPA2B1 (AUC=0.600)
LRPPRC (AUC=0.602)
METTL16 (AUC=0.421)
WTAP (AUC=0.647)
YTHDF3 (AUC=0.619)
ZC3H13 (AUC=0.698)
RiskScore (AUC=0.776)
FXR2 (AUC=0.343)
G3BP1 (AUC=0.460)
IGF2BP1 (AUC=0.627)
RBMX (AUC=0.503)
YTHDC2 (AUC=0.470)
YTHDF1 (AUC=0.414)
ZC3H13 (AUC=0.711)
Log Lambda
Coefficients
Partial Likelihood Deviance
Log(λ)
c
d
Points
Age
HPV_Status
NEGATIVE
POSITIVE
Grade
Stage
ZC3H13
RiskScore
Total Points
2−year survival
3−year survival
5−year survival
e
f
HNRNPA2B1
LRPPRC
G3BP1
WTAP
FMR1
YTHDF3
METTL16
ZC3H13
IGF2BP1
YTHDC2
FXR2
RBMX
YTHDF1
high risk
low risk
Risk score
Patients (increasing risk socre)
Dead
Alive
Recurrence
Recurrence−free
Recurrence−free survival time (years)

Fig.S4

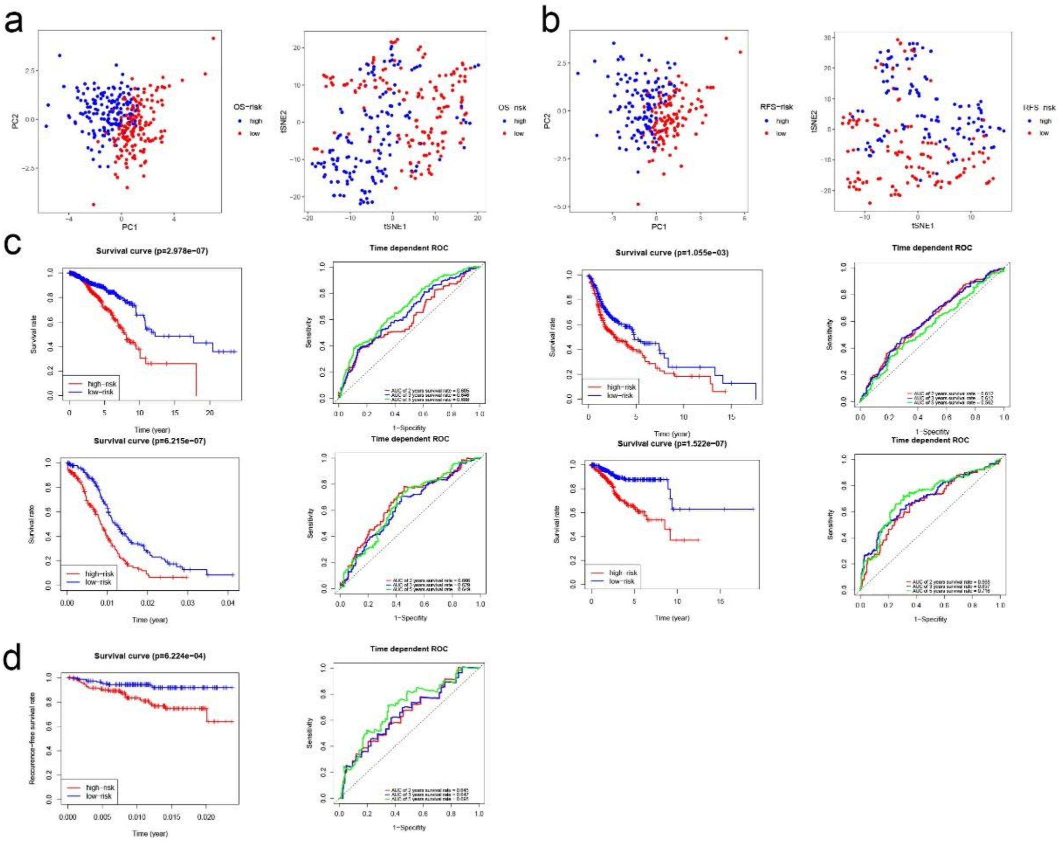
a
b
c
d
PC1
PC2
OS−risk
high
low
tSNE1
tSNE2
RFS−risk
Survival curve (p=2.978e−07)
Survival curve (p=1.055e−03)
Survival curve (p=6.215e−07)
Survival curve (p=1.522e−07)
Survival curve (p=6.224e−04)
Time dependent ROC
Survival rate
Recurrence−free survival rate
Time (year)
high−risk
low−risk
Sensitivity
1−Specifity

Fig.S5

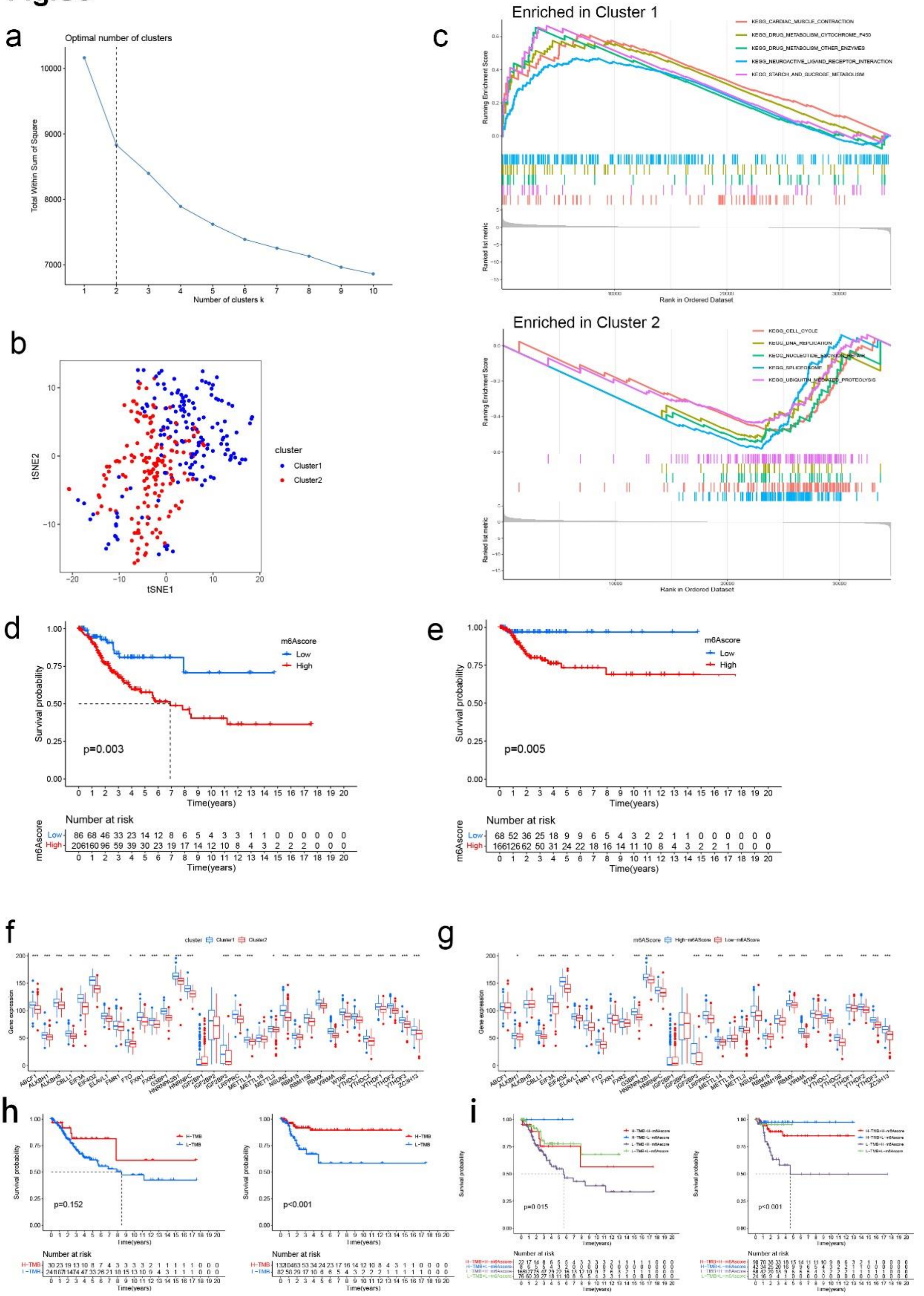
a
Optimal number of clusters
Total Within Sum of Square
Number of clusters k
b
tSNE1
tSNE2
cluster
Cluster1
Cluster2
c
Enriched in Cluster 1
KEGG_CARDIAC_MUSCLE_CONTRACTION
KEGG_DRUG_METABOLISM_CYTOCHROME_P450
KEGG_DRUG_METABOLISM_OTHER_ENZYMES
KEGG_NEUROACTIVE_LIGAND_RECEPTOR_INTERACTION
KEGG_STARCH_AND_SUCROSE_METABOLISM
Running Enrichment Score
Ranked list metric
Rank in Ordered Dataset
Enriched in Cluster 2
KEGG_CELL_CYCLE
KEGG_UBIQUITIN_MEDIATED_PROTEOLYSIS
d
m6Ascore
Low
High
Survival probability
p=0.003
Time(years)
Number at risk
e
p=0.005
f
cluster
Cluster1
Cluster2
Gene expression
g
m6AScore
High-m6AScore
Low-m6AScore
h
H-TMB
L-TMB
p=0.152
p<0.001
i
p=0.015
p<0.001

Fig.S6

a

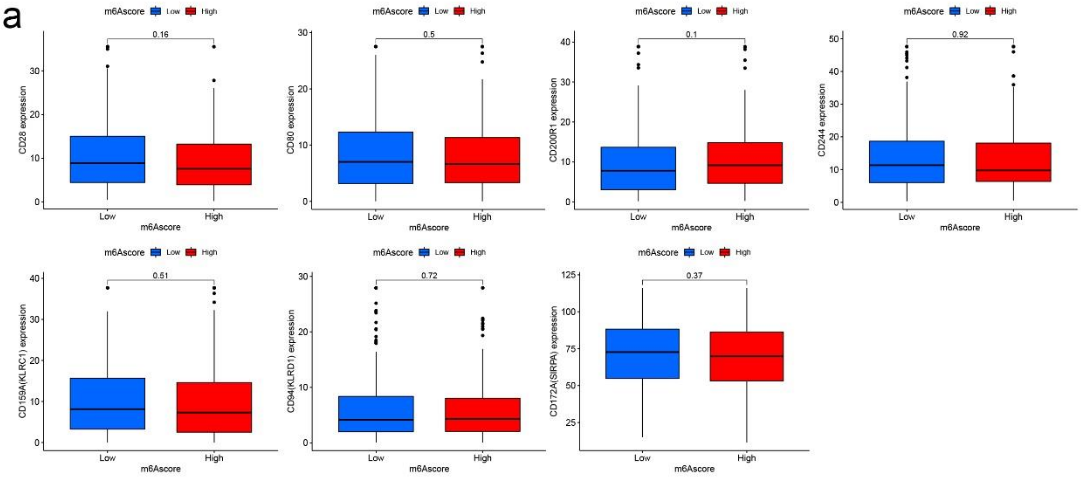
m6Ascore Low High
0.16
CD28 expression
0.5
CD80 expression
0.1
CD200R1 expression
0.92
CD244 expression
0.51
CD159A(KLRC1) expression
0.72
CD94(KLRD1) expression
0.37
CD172A(SIRPA) expression
Low
High
m6Ascore

Fig.S7

a

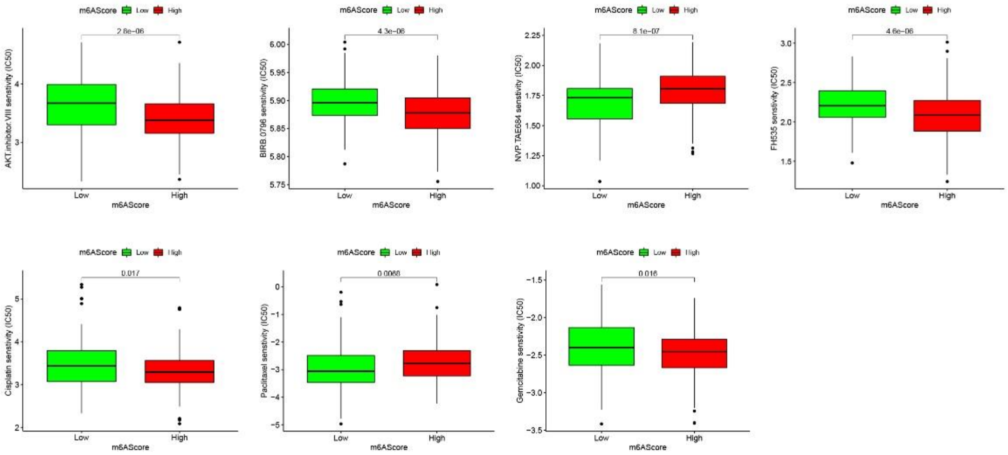
m6AScore Low High
2.8e−06
AKT.inhibitor.VIII sensitivity (IC50)
BIRB.0796 sensitivity (IC50)
4.3e−06
NVP.TAE684 sensitivity (IC50)
8.1e−07
FH535 sensitivity (IC50)
4.6e−06
Cisplatin sensitivity (IC50)
0.017
Paclitaxel sensitivity (IC50)
0.0068
Gemcitabine sensitivity (IC50)
0.016
Low
High
m6AScore

2 Supplementary Figures Legends

Fig.S1 Landscape of 33 m^{6}A regulators

a) The protein-protein interaction (PPI) network exhibited the interactions between regulatory factors. (minimum required interaction score > 0.4) b) The CNV variation frequency of 33 regulators in CESC patients. C) Correlation analysis between regulators based on TCGA-CESC, GSE63514 and GSE6791 cohorts (|R| > 0.3 and *P* < 0.05). d) Box plots showed the expression comparison between CC cases and normal control from TCGA-CESC, GSE63514 and GSE6791 cohorts.

Fig.S2 Steps of Diagnostic Model Construction and Key m^{6}A Regulators Identification

a) The process used to select the optimal model using the largest value. The final value used for the model was mtry = 2. b) The process of tree number determination with corresponding error in random forest fitting process for CC diagnosis. c) The process used to select the optimal model using the largest value for SVM model fitting. The final values used for the model were sigma = 0.01744768 and C = 8. d) Heatmap of ten key regulators for following ANN fitting. e) Establishment of CC diagnosis model with ten key m^{6}A factor as input layer based on ANN method.

Fig. S3 Steps of Prognostic Model Construction and Model Evaluation

a-b) The fitting process of LASSO-Cox regression for both OS (A) and RFS (B). The ROC curve demonstrated the ability of each factor to predict prognostic outcomes individually. c-d) The nomogram prognosis prediction model containing Age, HPV_Status, Grade, Stage, and risk for both OS (c) and RFS (d). e-f) Distribution of the riskscore, survival status, and the relative expression of candidate genes among CC patients for both OS (e) and RFS (f).

Fig. S4 Validation of Prognostic Model in HPV-related Cancers and Major Gynecologic Tumors

a-b) Different risk groups were identified based on prognostic risk scores and visualized by PCA and t-SNE for OS (A) and RFS (B). c) Kaplan-Meier (KM) survival curves and ROC curves showed a reasonable discriminative effect of OS prognostic model in BRCA, HNSC, OV and UCEC. d) The RFS prognostic model was validated by GSE4401, a CC dataset.

Fig. S5 Characteristic Exploration of Clusters and Immune Analysis Based on M^{6}AScore

a-b) Consensus clustering for k = 2 based on m^{6}A regulators (a) and visualization by t-SNE plot (b). c) GSEA analysis of patients in two clusters. d-e) OS (d) and RFS (e) KM curves for patients in different clusters. f-g) Expression level comparison of m6A regulators between clusters (f) and m6AScore group (g). h) OS and RFS related KM curves for CC patients with different TMB level. i) KM analysis with survival status or recurrence status as outcome variables. Groups were divided into different grades of TMB and m^{6}AScore combinations.

Fig.S6 Immune Checkpoints Expression between Low- and High-m6AScore Groups

a) Immune checkpoints CD28, CD80, CD200R1, CD244, CD159A, CD94 and CD172A expression between low- and high-m6AScore groups.

Fig. S7 Analysis of Treatment Strategies for Diagnosis and Prognosis based on m^{6}AScore

a) The box plots showed seven chemotherapeutics screened by estimating IC50 in low- and high-m^{6}AScore groups. The seven drugs include chemotherapy drugs clinically used to block oncogenic pathways (AKT.inhibitor.VIII, BIRB.0796, NVP.TAE684 and FH535) and CC chemotherapy drugs (Cisplatin, Paclitaxel and Gemcitabine).

3 Supplementary Tables

Table. S1 Details of the 33 regulatory factors used in this study

Gene	Alias	Ensembl ID	Chromosome Position
ABCF1	ABC27, ABC50	ENSG00000204574.12	chr6:30571376-30597179
ALKBH1	ABH, ABH1, ALKBH, alkB, hABH	ENSG00000100601.9	chr14:77672404-77708020
ALKBH5	ABH5, OFOXD, OFOXD1	ENSG00000091542.8	chr17:18183078-18209954
CBLL1	HAKAI, RNF188	ENSG00000105879.11	chr7:107743697-107761667
EIF3A	EIF3, EIF3S10, P167, TIF32, eIF3-p170, eIF3-theta, p180, p185	ENSG00000107581.12	chr10:119033670-119080823
EIF4G2	AAG1, DAP5, NAT1, P97	ENSG00000110321.15	chr11:10797050-10809110
ELAVL1	ELAV1, HUR, Hua, MelG	ENSG00000066044.13	chr19:7958579-8445041
FMR1	FMRP, FRAXA, POF, POF1	ENSG00000102081.13	chrX:147911951-147951125
FTO	ALKBH9	ENSG00000140718.18	chr16:53703963-54121941
FXR1	FXR1P	ENSG00000114416.17	chr3:180868141-180982753
FXR2	FMR1L2, FXR2P	ENSG00000129245.11	chr17:7591230-7614871
G3BP1	G3BP, HDH-VIII	ENSG00000145907.14	chr5:151771045-151812785
HNRNPA2B1	HNRNPA2, HNRNPB1, HNRPA2, HNRPA2B1, HNRPB1, IBMPFD2, RNPA2, SNRPB1	ENSG00000122566.20	chr7:26189927-26201529
HNRNPC	C1, C2, HNRNP, HNRPC, SNRPC	ENSG00000092199.17	chr14:21209136-21269494
IGF2BP1	CRD-BP, CRDBP, IMP-1, IMP1, VICKZ1, ZBP1	ENSG00000159217.9	chr17:48997412-49055650
IGF2BP2	IMP-2, IMP2, VICKZ2	ENSG00000073792.15	chr3:185643739-185825056
IGF2BP3	CT98, IMP-3, IMP3, KOC, KOC1, VICKZ3	ENSG00000136231.13	chr7:23310209-23470467
LRPPRC	CLONE-23970, GP130, LRP130, LSFC	ENSG00000138095.18	chr2:43886508-43996005
METTL14		ENSG00000145388.14	chr4:118685368-118715433
METTL16	METT10D	ENSG00000127804.12	chr17:2405562-2511891
METTL3	IME4, M6A, MT-A70, Spo8	ENSG0000	chr14:2149813

		0165819.11	3-21511375
NSUN2	MISU, MRT5, SAKI, TRM4	ENSG0000<br>0037474.14	chr5:6599239-<br>6633291
RBM15	OTT, OTT1, SPEN	ENSG0000<br>0162775.14	chr1:11033850<br>6-110346681
RBM15B	HUMAGCGB, OTT3	ENSG0000<br>0259956.1	chr3:51391268<br>-51397908
RBMX	HNRNPG, HNRPG, RBMXP1, RBMXRT, RNMX, hnRNP-G	ENSG0000<br>0147274.14	chrX:1368480<br>04-136880764
VIRMA	KIAA1429, MSTP054, fSAP121	ENSG0000<br>0164944.11	chr8:94487693<br>-94553529
WTAP	Mum2	ENSG0000<br>0146457.14	chr6:15972558<br>5-159756319
YTHDC1	YT521, YT521-B	ENSG0000<br>0083896.12	chr4:68310387<br>-68350089
YTHDC2	CAHL	ENSG0000<br>0047188.15	chr5:11351368<br>3-113595285
YTHDF1	C20orf21	ENSG0000<br>0149658.17	chr20:6319542<br>9-63216234
YTHDF2	CAHL, HGRG8, NY-REN-2	ENSG0000<br>0198492.14	chr1:28736621<br>-28769775
YTHDF3		ENSG0000<br>0185728.16	chr8:63168553<br>-63212786
ZC3H13	KIAA0853	ENSG0000<br>0123200.16	chr13:4595446<br>5-46052759

Table.S2 Details of human tissue samples used for qPCR assays

ID	Age	Alive_status	Time_of_first_diagnosis	HPV_status	Menopause_status	Stage_and_tumor_type
Participant1	41	Alive	2020/12/11	Positive	No	IA2
Participant2	30	Alive	2021/5/8	Positive	No	carcinoma in situ
Participant3	43	Alive	2021/6/30	Positive	No	carcinoma in situ
Participant4	53	Alive	2021/7/30	Negative	Yes	IIA1
Participant5	63	Alive	2020/11/4	Positive	Yes	IA1
Participant6	58	Alive	2021/5/25	Positive	Yes	carcinoma in situ
Participant7	22	Alive	2020/11/19	Positive	No	Ib1
Participant8	40	Alive	2021/6/25	Positive	No	IB1
Participant9	43	Alive	2021/10/20	Negative	No	IIIC1
Participant10	44	Alive	2020/12/15	Positive	No	Ib2
Participant11	51	Alive	2020/12/14	Positive	Yes	IB2
Participant12	61	Alive	2021/6/16	Negative	Yes	IB1
Participant13	57	Alive	2021/9/28	Negative	Yes	IIa1
Participant14	36	Alive	2020/12/19	Positive	No	carcinoma in situ
Participant15	43	Alive	2020/12/9	Positive	No	IB1
Participant16	43	Alive	2021/4/16	Positive	No	IA2
Participant17	41	Alive	2021/1/22	Positive	No	carcinoma in situ
Participant18	46	Alive	2020/11/11	Positive	No	carcinoma in situ
Participant19	43	Alive	2021/10/20	Positive	No	mixed adenocarcinoma, IIIC1
Participant20	43	Alive	2020/12/10	Positive	No	IB1

Table.S3 Primer sequences of key signatures for qPCR

GeneSymbol	Premier_Sequence	
GAPDH	GCACCGTCAAGGCTGAGAAC	Forward
	GGATCTCGCTCCTGGAAGATG	Reverse
ZC3H13	GTGCCGTAACTGGCTGAAGA	Forward
	CCTTTACCACGAGGTGAAGGG	Reverse
YTHDF3	CTTAAACCCAAGGGCAATGT	Forward
	TGGAGCCTTTACCACTGACC	Reverse
RBM15	CTGCCTGAGGAGAGTGGAGGAC	Forward
	CGGCTACTGCTCAATTCTGGACTG	Reverse
METTL3	AGTGACAGCCCAGTGCCTAC	Forward
	GCTTTCTACCCCATCTTGAGTG	Reverse
CBLL1	GCAGCGAATTGAGCAGTGTA	Forward
	AGTGAAGCACGGGTAACAGG	Reverse
NSUN2	ATTGCGAATGATGTGGACAA	Forward
	TCATAGTGCCGTCTCCACTG	Reverse
RBMX	AAGGATGCAGCCAGAGACATGAATG	Forward
	CTCTACGAGAGGGCAGCGGTTC	Reverse
HNRNPA2B1	TGGCTGCAAGACCTCATTC	Forward
	CGCCAACAAACAGCTTCTTC	Reverse
